# Differential Microbial Composition and Interkingdom Interactions in the Intestinal Microbiota of Holstein and German Simmental × Holstein Cross F1 Calves: A Comprehensive Analysis of Bacterial and Fungal Diversity

**DOI:** 10.3390/microorganisms12030486

**Published:** 2024-02-28

**Authors:** Shanshan Nan, Jiacheng Li, Yu Kuang, Jiaqi Feng, Hailiang Wang, Junli Niu, Yanyan Wu, Wenju Zhang, Cunxi Nie

**Affiliations:** College of Animal Science and Technology, Shihezi University, North Street 5, Shihezi 832000, China; 18016862099@163.com (S.N.); 20212013017@stu.shzu.edu.cn (J.F.);

**Keywords:** calf breed, gut microbiota, high-throughput sequencing

## Abstract

Calf intestines are colonized by rich and complex microbial communities, playing a crucial role in animal physiology, metabolism, nutrition, and immune function. In this study, we provide insight into the composition of fecal microbial bacteria and fungi, respectively, as well as the cross-kingdom interactions. We investigated the intestinal microbiota of different breeds of calves by characterizing the bacterial and fungal communities in the rectal feces of Holstein calves and German Simmental × Holstein cross F1 generation (GXH) using 16S rRNA and ITS amplicon sequencing techniques. PICRUSt2 (version 2.2.0) were used to determine microbial diversity and function and explore the reasons why Holstein calves are more susceptible to diarrhea. The results revealed no significant difference in the diversity of fecal microbiota among the groups (*p* > 0.05). We identified *Firmicutes, Bacteroidetes,* and *Proteobacteria* as the dominant bacterial phyla in the fecal bacterial communities of the two breeds of calves. *Ascomycota* and *Basidiomycota* play important roles in the fungal community but differ in relative abundance. *Bacteroides* was the dominant genus at the group level for calf fecal microbiota in both breeds. The relative abundance of *Prevotella*, *Escherichia*-*Shigella*, *Peptostreptococcus*, and *Butyricicoccus* was higher in Holstein calves, and the relative abundance of *Faecalibacterium*, *Megamonas*, *Butyricicoccus*, and *Alloprevotella* was lower than GXH group. *Aspergillus* and *Cladosporium* were the dominating genera of fecal fungi in both groups of calves. LEfSe analysis revealed 33 different bacteria and 23 different fungi between the two groups, with more differential strains found in GXH. In addition, the feces fungi-bacteria interkingdom interactions varied among breeds. Thus, the composition and structure of bacterial and fungal communities in calf feces varied by breed, indicating a potential association between breed and microbial communities. We also found differences in the network between bacterial–fungal kingdoms. We explain the reasons for Holstein calves being more prone to diarrhea. This indicated that breed makes differences in calf diarrhea rates by influencing gut microbial composition and interactions.

## 1. Introduction

Young animals are colonized by millions of microbiota in their gastrointestinal tract immediately after birth [1]. Microbiota, including bacteria, fungi, viruses, protozoa, and archaea, collectively form the rich and complex microbial micro-ecosystem known as the gut microbiome [2]. The gut microbiome interacts with the host to maintain gut health and plays a key role in host nutrient digestion, energy harvesting, immune regulation, and disease development [3]. Imperfect intestinal function in newborn calves leads to high morbidity and mortality from diarrhea, which is often associated with intestinal microorganism disturbances [4]. The gut microbiome can influence the overall immune response of the gut by producing metabolites (e.g., SCFAs) that stimulate intestinal epithelial cells to activate anti-inflammatory pathways or increase the expression of major tight junction proteins [5]. Thus, the composition of the early gut microbiome had a potential impact on the healthy growth of calves [6].

The intestinal microbiota is affected by both environmental (diet, medicine, geography) and host (genetic background, gender, and age) factors [7]. Evidence suggests that host genetics plays a key role in determining the composition and structure of the gastrointestinal bacterial community [8]. The formation of different microbial communities in the animal gut through maternal inheritance is associated with multiple developmental and pathogenic processes [9,10]. Fan et al. [11] evaluated the effect of host genetics on hindgut microbiota development in 3-month-old cattle. Cattle with varying breed compositions (100% Angus, 100% Brahman, and multi-breed Angus–Brahman) were raised and fed identically under the same environmental conditions. Results indicated that host genetics shapes the gut microbiota in the early life stages, which may impact animal growth and health. In addition, Imhann et al. [12] proposed that the interaction between host genetics and the gut microbiota underlie the pathogenesis of inflammatory bowel disease. Therefore, for the growth of healthy calves, it is crucial to obtain a balanced intestinal microbiota via maternal inheritance [13].

Considering the impact of host genetics on microbial communities, particularly in early life stages [11], this study focuses on GXH, the F1 generation of the German Simmental × Holstein cross, known for its advantageous traits in both dairy and meat cattle. This crossbreeding approach can enhance growth performance and accelerate body mass growth in crossbred offspring. Compared with Holstein cattle, GXH demonstrated evidently improved performance, milk fat and milk protein rates were significantly higher, resistance to adversity was significantly enhanced, and overall breeding efficiency was increased. In pursuit of understanding how breed influences the composition of intestinal bacterial and fungal ecosystems, this study employs 16S rRNA and ITS sequencing to characterize microbial communities in the intestines of calves from different breeds. By unraveling the differences between the intestinal microbiota of Holstein and GXH calves, this investigation aims to shed light on the reasons behind the higher susceptibility of Holstein calves to diarrhea, contributing valuable insights for promoting the health and well-being of these young animals [14].

## 2. Materials and Methods

### 2.1. Animals and Experimental Procedure

This study collected samples from 70 calves of similar age (14–32 days), 35 Holstein and 35 GXH calves (German Simmental × Holstein Cross F1 Calves), with an average weight of 43.30 ± 5.48 kg and 37.20 ± 5.12 kg, respectively. The test calves were obtained from Tianshan Military Reclamation Animal Husbandry Co., Ltd., Shihezi, Xinjiang (Located at 44°12′17″ N, 86°4′3″ E). All calves were reared in the same way. The calves were placed in individual pens (1.2 m × 1.5 m) and fed 4 L of pasteurized milk (8 L/day for 14–49 days) at 8:00 and 20:00 daily. The pasteurized milk provided herein was analyzed with Delta Combi Scope 300 hp milk composition analyzer (Delta Instruments, The Netherlands) and contained 12.1% dry matter, 3.89% fat, 3.23% protein, and 4.85% lactose. Pasteurized milk was given to each calf in a separate open bucket, which was cleaned after each feeding and dripped dry before the next meal. The calves were allowed to drink and consume the concentrate (Zhengda, Xinjiang, China) ad libitum for the duration of the trial. The composition of the concentrate is shown in Table 1. No antimicrobial drugs were used before or during the trial. The composition of the starter concentrate is shown in Table 1.

### 2.2. Sample Collection

The health status of each sampled calf was assessed by clinical examination. Healthy calves had to be free of diarrhea, while sick calves showed signs of abnormal fecal consistency at the time of sampling. Feces were evaluated using the scoring system (1 to 4) published by Larson et al. [15], where scores of 3 and 4 were classified as diarrhea. If the shape of the feces was normal but not hard, and the shape changed slightly after falling on the ground, it was marked as 1 point. If the feces were soft, piled up, and it was difficult to maintain their shape, it was marked as 2 points. If the feces were in the form of pancakes or ointments that were easy to apply, the score was 3 points. Finally, if it had the consistency of water and showed solid–liquid separation, it was marked as 4 points. The diarrhea rate was calculated at the completion of the evaluation. All animals were restrained in a veterinary crush before sampling to ensure the safety of animals. After sampling, all experimental animals were safely released. Sampling personnel removed feces from the rectum using sterile gloves, and the samples were immediately placed in sterile cryostorage tubes, placed in liquid nitrogen, and stored at −80 °C until DNA extraction.

### 2.3. DNA Extraction and PCR Amplification

Total DNA was extracted from 0.2 g of feces using the E.Z.N.A.^®^ Stool DNA Kit (Omega Bio-tek, Norcross, GA, USA) according to the method described by Rosa et al. [16]. To further ensure the quality of the extracted total genomic DNA, 1% agarose gel electrophoresis and NanoDrop2000 (Thermo Scientific Inc., Waltham, MA, USA) were used to assess the integrity and concentration of the extracts, respectively. The bacterial 16S rDNA (338F: ACTCCTACGGGAGGCAGCA and 806R: GGACTACHVGGGTWTCTAAT) and fungal ITS (ITS5F: GGAAGTAAAAGTCGTAACAAGG and ITS2R: GCTGCGTTCTTCATCGATGC) genes primers included were used to amplify the V3/V4 hypervariable and ITS2 regions, respectively. PCR products from the same samples were mixed and recovered in 2% agarose gels, purified using AxyPrep DNAGel Extraction Kit (Axygen Biosciences, Union City, CA, USA), detected by 2% agarose gel electrophoresis, and detected and quantified by QuantusTM Fluorometer (Promeg, Madison, WI, USA). Library construction and sequencing were performed using the NEXTFLEX Rapid DNA-Seq Kit t (Bioo Scientific, Austin, TX, USA) and the Illumina MiSeq PE300 platform (Illumina, San Diego, CA, USA), respectively.

### 2.4. Bioinformatics Analysis

The raw sequenced sequences were quality-controlled using Fastp (version 0.19.6, https://github.com/OpenGene/fastp, accessed on 5 May 2023) and spliced using Flash (version 1.2.7, http://www.cbcb.umd.edu/software/flash, accessed on 12 May 2023). The sequences were operational taxonomic units (OTUs) clustered, and chimeras were removed based on a 97% similarity threshold using Uparse (version 7.1, http://www.drive5.com/uparse/, accessed on 20 May 2023). Based on the Silva 16S rRNA database (version 138, https://www.arb-silva.de/, accessed on 22 May 2023) and UNITE ITS fungi database (version 8.0, https://unite.ut.ee/, accessed on 25 May 2023). The taxonomy of each OTU representative sequence was performed using an RDP classifier (version 2.11, http://rdp.cme.msu.edu/, accessed on 1 June 2023), and a confidence threshold of 0.7 was set to obtain the taxonomy annotation results for community composition analysis at different taxonomic levels. (A cutoff of 0.7 was used because of the high percentage of sequences from our datasets that were assigned as unclassified when using the recommended threshold of 0.8; this may be due to the clusters in our datasets being too novel for the training dataset in the RDP classifier). The results are presented as bar charts and pie charts for visualization. The OTU abundance information was normalized using the sequence number criteria corresponding to samples with the least number of sequences. The intestinal microbial alpha diversity was calculated based on the relative abundance distribution of OTUs in each sample. Beta diversity was calculated based on weighted UniFrac distances to assess the differences and similarities across samples and groups. The linear discriminant analysis (LDA) effect size (LEfSe) was used to identify bacterial taxa that differed between cultivars in the intestinal bacterial and fungal communities. Taxonomy with an LDA score > 2.0 was considered an important biomarker for each group.

### 2.5. Statistical Analysis

The data were statistically analyzed using SPSS 22.0 (SPSS Inc., Chicago, IL, USA). Statistical significance was set at *p* < 0.05, and these values were presented as SD ± mean. Cytoscape (version 3.9.0) was used to graph Network. ORIGIN 2021 (OriginLab Corporation, Northampton, MA, USA) was used for Spearman’s analysis and graph preparation.

### 2.6. Predicted Function of the Microbiome

To reveal the potential metabolic capacity of bacterial communities, we used PICRUSt 2 (version 2.2.0, http://picrust.github.io/picrust/, accessed on 15 June 2023) to predict the function of the gut microbiota based on OTU tables [17]. Functional information on the species corresponding to OTU was obtained by aligning the available 16S rRNA sequencing data with the Greengenes database.

## 3. Results

### 3.1. Birth Traits and Diarrhea Incidence in Various Cattle Breeds

As shown in Table 2, the body height, body oblique length, and body weight of GXH calves at birth were significantly higher, and the rate of diarrhea incidence was lower than Holstein calves. The tube circumference was higher than Holstein steers; however, the difference was not significant (*p* > 0.05).

### 3.2. Bacterial Diversity in Fecal Microbiota of Different Breeds of Calves

#### 3.2.1. Sequencing Statistics and Bacterial Diversity

**Figure 1:**
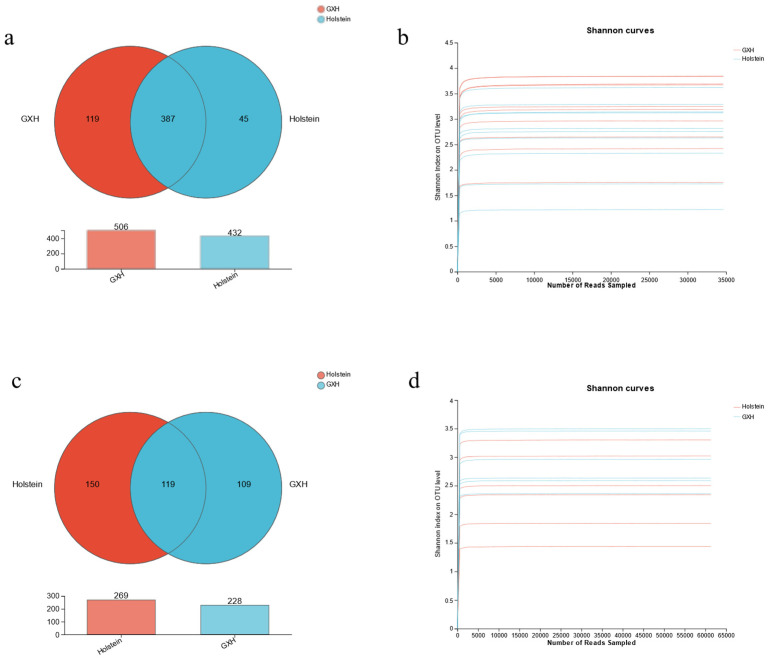
Sequencing data feasibility analysis and OTUs distribution. (**a**) Bacterial rarefaction curves for all samples. (**b**) Distribution of bacterial OTUs in different samples. (**c**) Fungal sparsity curves of all samples. (**d**) Distribution of fungal OTUs in different samples.

In this study, amplicon sequencing was performed on 20 fecal samples from 10 Holstein and 10 GXH calves. A total of 1,410,903 raw sequences were collected from the V3/V4 region. After sequence filtering, 1,188,901 complete, high-quality reads were obtained from all samples, with an average read of 59,445 reads per sample from the bacterial population. Following taxonomic assignment, a total of 551 bacterial OTUs were identified based on 97% nucleotide sequence similarity (Figure 1a). The rarefaction curves for each sample showed a flat trend, indicating that this sequencing depth captured most of the organisms present in the sample (Figure 1b).

**Figure 2:**
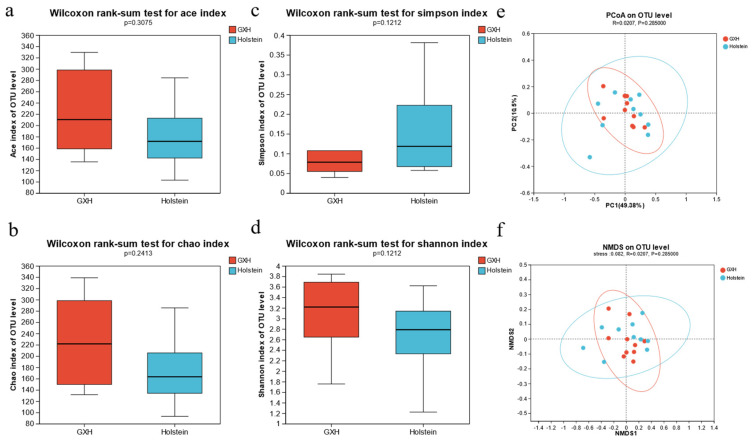
Differences in fecal bacterial diversity among different breeds of calves. Bacterial alpha diversity can be evaluated by ACE (**a**), Chao (**b**), Simpson (**c**), and Shannon (**d**). Principal coordinate analysis (PCA) (**e**) and weighted UniFrac distance based on NMDS analysis (**f**) of fecal bacterial communities.

To obtain more accurate alpha diversity results, we reduced the sequences to 34,642 per sample and used this sequence for microbial diversity, composition, and structure analysis. Alpha diversity was calculated from the rectal fecal microbial communities of calves of the different breeds. There were no significant differences in the Shannon, Simpson, ACE, and Chao indices (*p* > 0.05), indicating that there were no significant differences in the richness and diversity of fecal microorganisms in the Holstein and GXH groups (Figure 2a–d).

Principal coordinate analysis (PCoA) was used to evaluate the similarity and variability of the samples between and within groups. The contributions of principal component 1 and principal component 2 of PCoA were 19.87% and 13.17%, respectively. PCoA scatter plots of the bacterial communities showed inconspicuous separation of individuals in the Holstein and GXH groups, indicating that the predominant composition of rectal fecal microbes is unaffected by the breed (Figure 2e). Non-metric multidimensional scaling was used to examine the community composition of rectal fecal microbiota in the two groups of calves. Non-metric multidimensional scaling analysis (NMDS) based on the weighted UniFrac distance matrix showed that samples from Holstein and GXH calves were close, indicating no significant difference in fecal bacterial community composition between the two groups (ANOSIM: weighted UniFrac, R = 0.021, *p* = 0.285; Figure 2f).

#### 3.2.2. Taxonomic Composition and Differences in Fecal Bacterial Community

**Figure 3:**
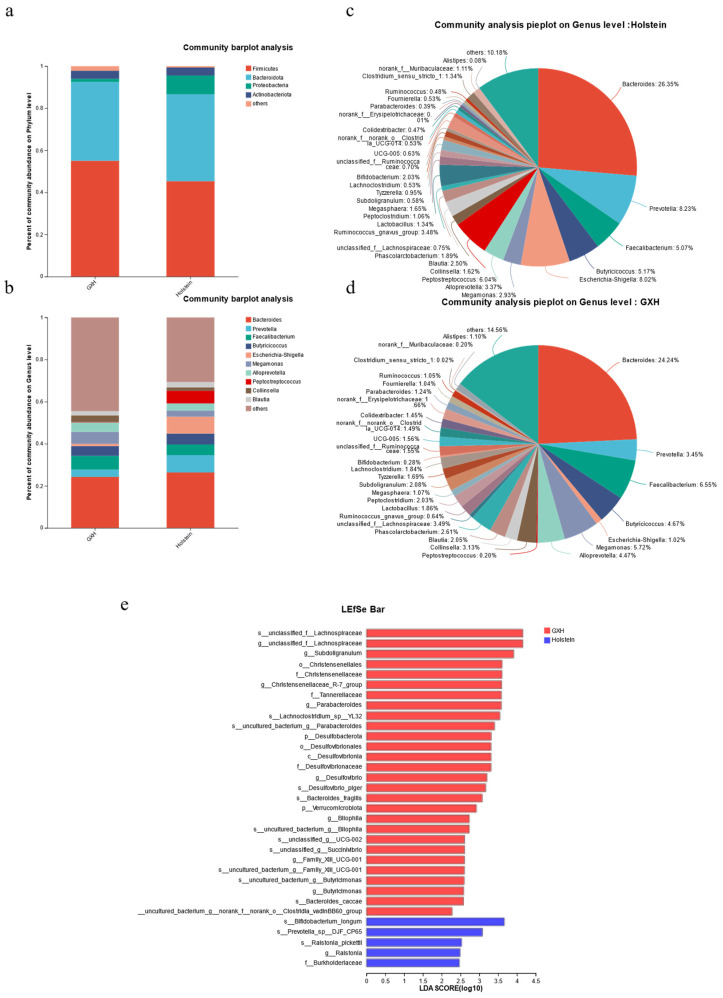
Difference analysis of intestinal bacteria between GXH group and Holstein. The bacterial composition at the phylum (**a**) and genus (**b**) levels. Pie charts at the genus level for the Holstein (**c**) and the GXH group (**d**). The differences in the relative abundance of bacteria between GXH and Holstein (**e**).

The relative proportions of dominant taxa at different taxonomic levels were determined by microbial taxon assignment, and differences in the abundance and composition of fecal bacteria between the two groups were observed. At the phylum level, 16 phyla were identified across 20 samples. *Firmicutes*, *Bacteroidota*, *Proteobacteria*, and *Actinobacteriota* were the four dominant phyla in the Holstein and GXH breeds, accounting for approximately 97% of the total taxonomic groups identified in all samples (Figure 3a). To further explore the influence of breed on taxonomic composition, 200 genera were detected in the calf rectal fecal microbiota. Of these identified genera, *Bacteroides* (26.35%, 24.24%) was the predominant bacterial genus in both breeds (Figure 3b). More than 53.79% of the total bacterial composition in the Holstein group (Figure 3c) was accounted for by *Prevotella* (8.23%), *Escherichia*-*Shigella* (8.02%), *Peptostreptococcus* (6.02%), and *Butyricicoccus* (5.17%). Meanwhile, *Faecalibacterium* (6.55%), *Megamonas* (5.72%), *Butyricicoccus* (4.67%), and *Alloprevotella* (4.47%) were present in large numbers in the GXH group, accounting for approximately 45.65% of the total taxonomic groups identified (Figure 3d).

The LEfSe analysis was used to compare microbiota differences between the Holstein and GXH groups. Of the 33 differential bacteria found, 28 were enriched in GXH and 5 were enriched in Holstein (Figure 3e and Supplemental Table S1), indicating that there were more differential bacteria in GXH.

#### 3.2.3. Functional Predictions of the Rectal Microbiota Using PICRUSt

**Figure 4:**
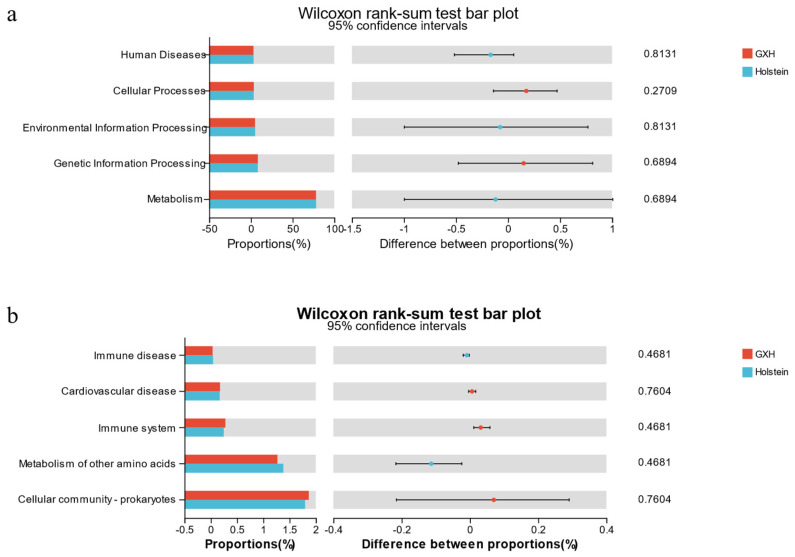
Differential analysis of function prediction in GXH and Holstein groups. Functional prediction analysis of 16S rRNA gene sequences of intestinal bacteria based on KEGG database. Pathways Level 1 (**a**) and Level 2 (**b**).

We used PICRUSt to predict the function of fecal microbiota in different breeds of calves and used NSTI to assess the accuracy of these predictions. Five biometabolic pathways were identified in the level 1 pathway, including metabolism, Genetic Information Processing, Environmental Information Processing, Cellular Processes, and Human Diseases (Figure 4a). The predicted secondary pathways primarily comprised 45 sub-functions with the most representative level 2 pathways being cellular community–prokaryotes and the metabolism of other amino acids (Figure 4b).

#### 3.2.4. Correlation Network Analysis

**Figure 5:**
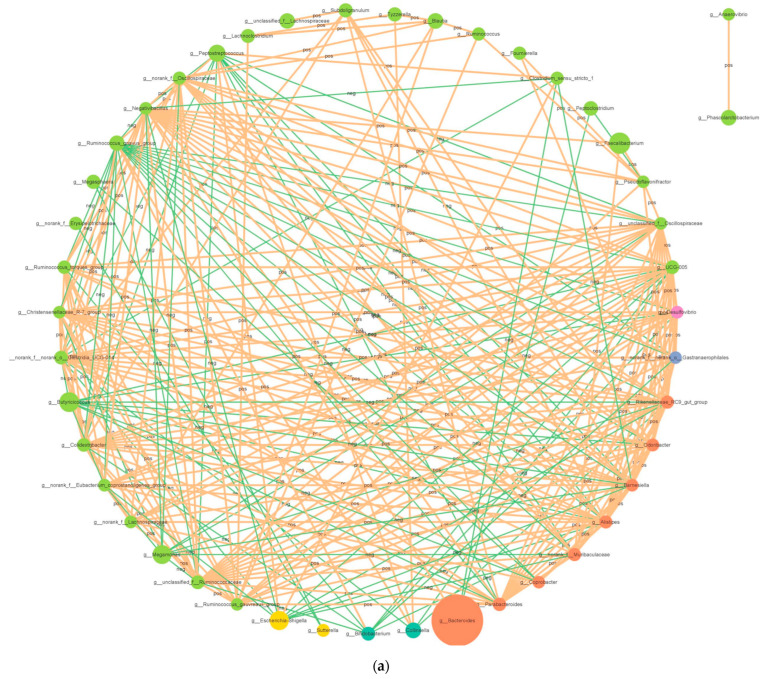

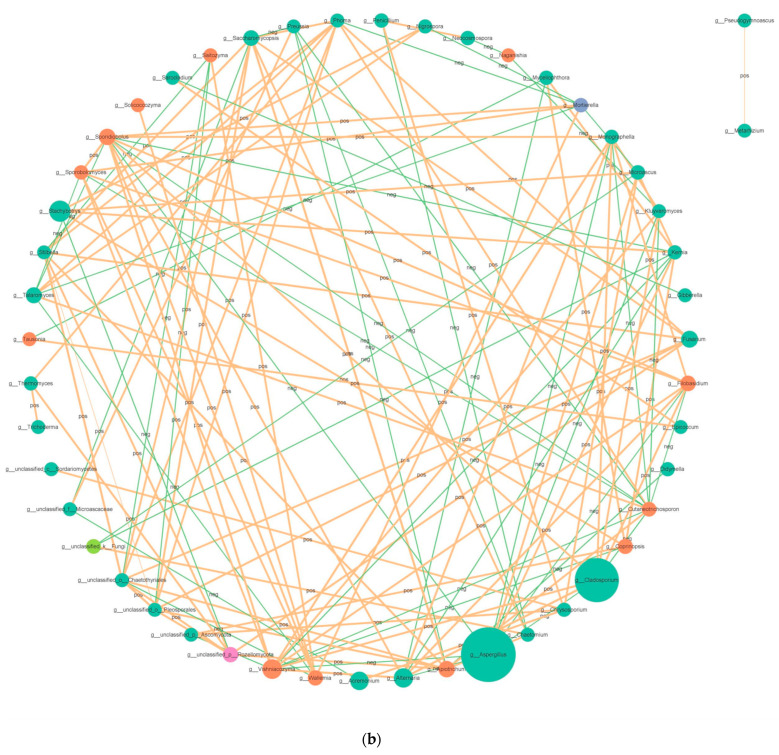
Correlation network reveals the correlation among the different bacterial (**a**) and fungal (**b**) genera. The circles with different colors indicate the names of bacterial and fungal genus, and their sizes represent relative abundance. The strength of correlation between two genera is positively correlated with the thickness of the line. The orange line indicates positive correlation, while the green line indicates negative correlation.

Network analysis was performed using the Cytoscape software (version 3.9.0) to elucidate the connections between different bacterial genera in the fecal microbiota (Figure 5a). The bacterial network consisted of 45 nodes and 270 edges. The results showed 195 positive and 75 negative correlations in the bacterial network analysis at the genus level. *g__Butyricicoccus* was positively correlated with *g_Ruminococcus _gnavus_group* (0.8445). *g_Peptostreptococcus* was negatively correlated with *g_norank_f_norank_o_Clostridia_UCG-014* (−0.7869), and *g_Alistipes* was negatively correlated with *g_Ruminococcus_gnavus_group* (−0.7556).

### 3.3. Fungal Diversity in the Fecal Microbiota of Different Breeds of Calves

#### 3.3.1. Sequencing Statistics and Fungal Diversity

Using the Illumina Nova sequencing platform, 12 fecal samples from Holstein and GXH calves were sequenced by ITS high-throughput sequencing. A total of 1,142,916 raw sequences were obtained, with an average of 95,243 sequences per sample, and 1,002,440 clean tags were obtained after quality control, with an average of 85,968 sequences per sample and an average length of 229 bp per sequence. The dilution curves of each sample tended to be flat (Figure 1c), indicating that the amount of data obtained from sequencing in this experiment covered most of the species in the samples and satisfied the conditions for subsequent microbial diversity analysis. The sequences were clustered into OTUs with a 97% similarity principle (Figure 1d), and a total of 378 OTUs were obtained, including 269 in the Holstein group and 228 in the GXH group. The two groups shared 117 OTUs, accounting for 30.96% of the total number of OTUs.

**Figure 6:**
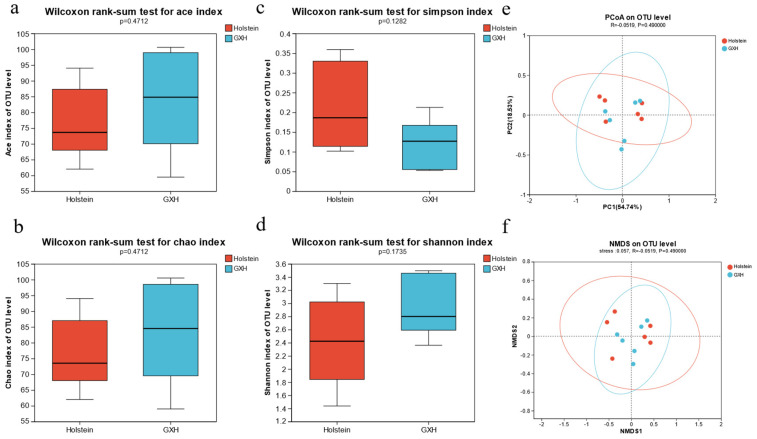
Differences in fecal fungal diversity among different breeds of calves. Fungal alpha diversity can be evaluated by ACE (**a**), Chao (**b**), Simpson (**c**), and Shannon (**d**). Principal coordinate analysis (PCA) (**e**) and weighted UniFrac distance based on NMDS analysis (**f**) of fecal fungal communities.

The results of alpha diversity analysis of fecal fungal microbiota are shown (Figure 6a–d), and the differences in Shannon, Simpson, ACE, and Chao indices of fecal fungal microbiota of calves were not significant between breeds (*p* > 0.05). To investigate the effect of breed on the composition of the fecal fungal community in calves, PCoA analysis based on weighted UniFrac distances (Figure 6e) and NMDS plots (Figure 6f) indicated an overlap between individuals of the two groups. ANOSIM showed a significant difference in the fungal community composition between the two groups (R = −0.052, *p* = 0.490), indicating that the composition of the fecal fungal community was not affected by the cultivar.

#### 3.3.2. Taxonomic Composition and Differences of Fecal Fungal Community

**Figure 7:**
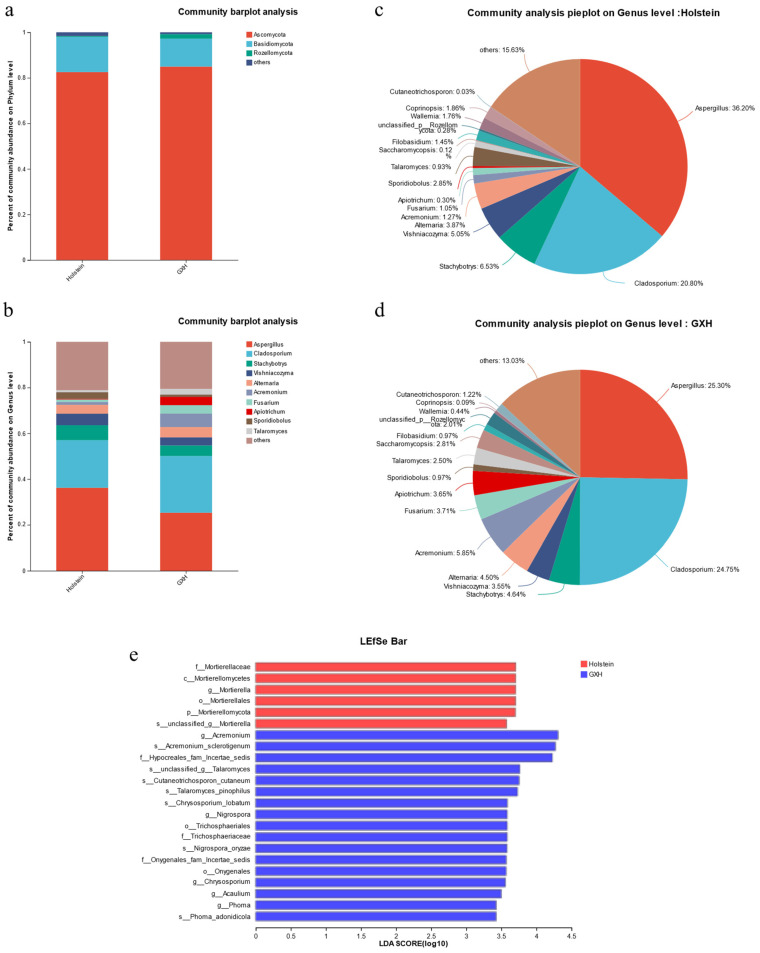
Differential analysis of intestinal fungi between GXH and Holstein groups. Fungal composition at the phylum (**a**) and genus (**b**) levels. Pie charts at the genus level for the Holstein (**c**) and the GXH group (**d**). The differences in the relative abundance of fungi between GXH and Holstein include (**e**).

The effective sequences obtained from sequencing were annotated at different taxonomic levels, and a total of 10 phyla, 30 classes, 68 orders, 126 families, 207 genera, and 307 species were identified. At the phylum level, the dominant phyla of fecal fungi in calves in the Holstein and GXH groups were mainly *Ascomycota* (82.51%, 84.92%), *Basidiomycota* (15.45%, 12.26%), *Rozellomycota* (0.38%, 2.01%), and others (1.66%, 0.81%). Among these, *Ascomycota* was the dominant phylum (Figure 7a).

*At the genus level, the top 10 genera were Aspergillus, Cladosporium, Stachybotrys, Vishniacozyma*, *Alternaria*, *Acremonium*, *Fusarium*, *Apiotrichum*, *Sporidiobolus*, and *Talaromyces* (Figure 7b). Among them, *Aspergillus* (36.20%, 25.30%) and *Cladosporium* (20.80%, 24.75%) were the dominant genera of fecal fungi in calves in the Holstein and GXH groups. Second, *Stachybotrys* (6.53%), *Vishniacozyma* (5.05%), *Alternaria* (3.87%), and *Sporidiobolus* (2.85%) accounted for more than 18.3% of the total fungal composition in the Holstein (Figure 7c), whereas, *Acremonium* (5.85%), *Stachybotrys* (4.64%), *Alternaria* (4.50%), and *Fusarium* (3.81%) were present in the GXH group (Figure 7d).

Microorganisms with significant differences in abundance at each taxonomic level were comparatively analyzed using LEfSe. The results shows 23 fungal microbiota (LDA score > 2.0) identified as biomarkers, 6 of which were found in Holstein cattle, including *g_Mortierella* and *s_unclassified_g_Mortierella*, and 17 were found in the GXH group, including *g_Acremonium*, *g_Nigrospora*, *g_Chrysosporium*, and *g_Acaulium*, *g_Phoma* (Figure 7e and Supplemental Table S2).

#### 3.3.3. Correlation Network Analysis

The fungal network comprised 39 nodes and 130 edges (Figure 5b). The results showed 84 positive and 46 negative correlations in fungal network analysis. *g_Aspergillus* was negatively correlated with *g_Alternaria* (−0.8601) and *g_Cladosporium* (−0.8322), and *g_Alternaria* was positively correlated with *g_Cladosporium* (0.9371).

### 3.4. Different Breeds Exhibited Variation in Bacterial–Fungal Associations

**Figure 8:**
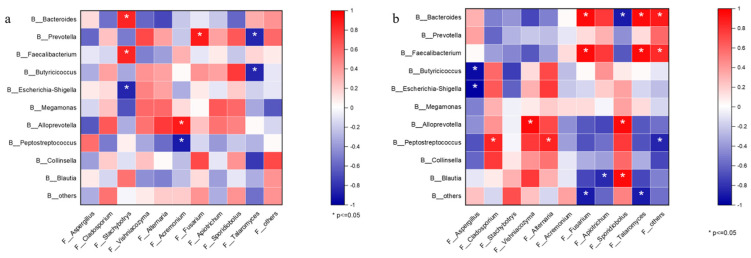
Bacterial–fungal correlations using Spearman’s correlation test in the two breeds. Specific bacterial–fungal correlation pattern in Holstein (**a**). Specific bacterial–fungal correlation pattern in GXH group (**b**). Positive values (red squares) indicate positive correlations, and negative values (blue squares) indicate inverse correlations. The shading of the square indicates the magnitude of the association; darker shades are more strongly associated than lighter shades. The sign (positive or negative) of the correlation was determined using Spearman’s method. * indicates *p* < 0.05.

To explore associations between bacteria and fungi, we examined the 10 genera with the highest relative abundance of bacterial–fungal correlations. Positive relationships linking *Stachybotrys* with *Bacteroides* and *Faecalibacterium* and negative correlations with *Escherichia–Shigell* were observed in Holstein calves (Figure 8a). Interestingly, the Spearman correlation was higher in GXH calves compared to Holstein. The relative abundance of *Fusarium*, *Talaromyces*, and *F__others* in GXH was positively correlated with the relative abundance of *Bacteroides* and *Faecalibacterium* and was negatively correlated with *B__others*. And there were more positive correlations between *Peptostreptococcus* and *Cladosporium*, *Alternaria*, and negative correlations with *F__others* (Figure 8b). In summary, these results indicated a complex relationship between bacteria and fungi in the microbiota, and we observed differences between the fungal–bacterial correlation analyzed in different calf breeds.

## 4. Discussion

Ruminants colonize trillions of microorganisms in the gut that interact with the host and co-evolve. Most microbes inhabiting the gut have profound effects on host physiology and nutrition, which play an integral role in host health [18]. Moreover, metabolites produced by gut microbes and host receptors act through critical interactions between the gut microbiota and different organs [19]. These interactions have profound impacts on the immunity, digestion and absorption, health, growth, and reproduction of the host animal [20]. Therefore, it is crucial to understand the symbiotic relationships between microorganisms and their hosts. However, most microbiome studies have focused on bacteria without considering the multinational nature of microbial ecosystems. The functional ecology of fungi, as an integral part of the microbiome, is poorly understood. In this study, we used 16S rDNA and ITS high-throughput sequencing to analyze and compare the differences in the fecal microorganisms of different breeds of calves, revealing the diversity of the gut microbial composition.

The gut microbiome is regulated by many factors, including growth environment, diet, and host genotype [21]. The structure and composition of the initial gut microbes of domestic animals are derived from the uterus [22] and are significantly influenced by the microbes in the birth canal [23]. We investigated the impact of breed on the fecal microbiota of calves living in the same environment but belonging to different breeds. Diversity analysis revealed no significant differences between the two breeds. However, analysis of species composition differences revealed that a small amount of microbiota differed. Thus, individual differences in early gut microbiota are primarily explained by genetic differences within the host.

Previous studies have shown that the fecal microorganisms of cattle are dominated by *Firmicutes*, *Bacteroidetes*, *Proteobacteria*, and *Actinobacteria* [24], which is consistent with the results of the present study. An investigation showed that the fecal microbiota of healthy individuals was dominated by *Bacteroidetes* and *Firmicutes* [25]. Brulc et al. [26] revealed that in herbivores, *Firmicutes* are primarily responsible for carbohydrate utilization, whereas *Bacteroidetes* are primarily responsible for cellulose catabolism. It plays a beneficial role in the microbial ecology of the ruminant gut. In this study, there were significant differences in F/B in the gut microorganisms of the different breeds, reaching an F/B value of 1.47 in the GXH group, which was higher than that of the Holstein group (F/B = 1.09). Several studies have shown a decrease in the F/B ratio and relative abundance of *Firmicutes* in numerous IBD cases [27]. Moreover, the phenomenon was present in healthy and diarrhea-ridden cattle gut microorganisms [28]. *Proteobacteria* are an important group of bacteria after *Firmicutes* and *Bacteroidetes*, and are mainly composed of pathogenic bacteria such as *Escherichia*, *Salmonella*, *Shigella genera*, and *Vibrio*. These bacteria can trigger inflammatory responses in animals by producing substances such as lipopolysaccharides and irritant flagellin [29]. In model species such as mice, supplementation with moderate amounts of *Proteobacteria* significantly reduced the severity of colitis and tended to correct the dysbiosis associated with colitis [30]. Harry Sokol et al. [31] hypothesized that metabolites secreted by *Proteobacteria* could block NF-κB activation and IL-8 production. In this study, the relative abundance of *Proteobacteria* was higher in the Holstein (8.92%) compared to the GXH group (1.53%). However, the excessive relative abundance of *Proteobacteria* is characteristic of dysbiosis in intestinal microorganisms [32]. Shin et al. [33] observed that a highly stable gut microbiota has a symbiotic interaction with the host immune system and suppresses the pathogenic bacterial population. The higher proportion of *Proteobacteria* in the intestine suggests their proliferation, indicating an unstable structure of the gut microbial community. This unstable structure has been observed in non-diseased states (e.g., neonatal diarrhea). Therefore, it predicts weaker resistance to diarrhea in Holstein calves.

Further analyses were performed at the genus level to gain more insight into the differences in the fecal microbes of the two breeds. *Prevotella* and *Butyricicoccus*, which were included in two groups, produce hydrolases that degrade plant polysaccharides and cellulose to promote host growth [34]. The present study found a higher relative abundance of *Prevotella* in Holstein calves compared to the GXH group. An increased relative abundance of *Prevotella* has been found to be associated with pro-inflammatory functions, and treatment of mice with *Prevotella* exacerbates colitis [35]. And *Escherichia–Shigella* is a dominant, highly virulent genus in the Holstein group, which produces toxins that can cross intestinal epithelial cells and induce inflammatory responses in animals [36]. This will lead to an increased chance of intestinal dysbiosis in Holstein calves, which is detrimental to gut health. In contrast, *Faecalibacterium*, the dominant genus in the gut of the GXH group, is a key marker of a healthy gut and has anti-inflammatory and immunomodulatory properties [37]. In another study, the relative abundances of *Faecalibacterium* and *Megamonas* in the intestines of frail individuals were lower than those of healthy individuals [38]. Therefore, we speculated that the higher diarrhea rate in Holstein calves was related to the community structure of the intestinal bacteria.

In the GXH group, the intestine was characterized by the presence of the genera *Subdoligranulum*, *Parabacteroides*, *Desutfovibrio*, and *Butyricimonas.* Previous studies found that *Subdoligranulum* was the most influential species in healthy individuals compared to patients with IBD, indicating its potential beneficial effects in managing IBD [39]. Furthermore, *Subdoligranulum* activates CD4+ T cells in the immune system of humans with inflammatory responses and promotes the IgA response of the body. Pathogenic microorganisms lose their ability to adhere to epithelial cells [40]. *Butyriconas* was reduced in the gut of patients with ulcerative colitis compared to healthy individuals [41], indicating the possibility of mediating intestinal inflammation. Brown et al. [42] explained that butyrate-producing bacteria, such as *Subdoligranulum* and *Butyricimonas*, enhanced epithelial barrier integrity by butyric acid-induced mucin synthesis and increased the expression of tight junction proteins [43]. To prevent intestinal inflammation caused by the overgrowth of harmful microbial species, it is important to maintain the integrity of the intestinal mucosal barrier [39]. However, *Desulfovibrio* was the main producer of H_2_S in the gastrointestinal tract. The diffusion of H_2_S released by bacteria into the intestinal epithelium causes intestinal sensitization or diarrhea [44]. Remarkably, *Desulfovibrio* produces acetic acid by consuming lactic acid, forming a symbiotic relationship with lactic acid-producing *Faecalibacterium*. This simultaneous presence will help the intestine produce more acetic acid to maintain intestinal health [45]. As a result, specific bacteria in the intestines of the GXH group maintained intestinal mucosal barrier integrity.

As the development of host diseases is often accompanied by disruptions in the dynamic balance of the gut microbiota, the intestinal microbiota plays an important role in promoting host health [46]. This perspective is widely accepted. Moya et al. [47] believed that different microorganisms perform similar functions and that redundancy occurs between the functions of the intestinal microorganisms. In this study, we found that although different cattle breeds possessed different gut microbiota structures, the bacterial communities exhibited similar biological functions [48]. By comparing the KEGG pathways, the fecal organisms of the rectal contents of the two groups of calves were found to be enriched in metabolism, Genetic Information Processing, Environmental Information Processing, Cellular Processes, and Human Diseases. Meanwhile, the immune system and immune disease functionally related KEGG pathways were enriched. Previous studies have indicated that the composition of the gut microbiota is related to the immune system status and health of the host [49], and the differences observed may suggest differences in immune function between the two breeds.

Similarly, fungi are important components of the gut microbiota and play important roles in gut function and host health [50]. Fungal communities in ruminants are responsible for lignocellulose breakdown. It promotes absorption and utilization through physical permeation and secretion of cell wall-degrading enzymes. The synthesis of metabolites, such as acetate and formate, plays a regulatory role in the intestine [51]. The fungal microbiota of both groups was dominated by *Ascomycota* and *Basidiomycota*. Interestingly, although the difference in fungal diversity between the two groups was not significant, the structure and proportion of microbiota changed. The observed higher *Basidiomycota/Ascomycota* ratio in Holstein (18.73%) compared to GXH (14.44%) is noteworthy, considering similar findings in patients with inflammatory bowel disease (IBD) [52]. This alteration may indicate a potential association with inflammation, further suggesting that Holstein calves could be more prone to symptoms like diarrhea. Previously, fungi have been shown to induce intestinal inflammation and increase intestinal mucosal permeability [53]. Therefore, Holstein calves are more prone to developing symptoms such as diarrhea. At the genus level, *Aspergillus* and *Cladosporium* are the main taxa. *Aspergillus* spores are ubiquitous and several *Aspergillus* cause major invasive diseases. Their relative abundance showed a strong positive correlation with diarrhea [54]. Specific *Aspergillus* spp. such as *Aspergillus amstelodami* and *Aspergillus fumigatus* can exacerbate colitis [55]. We observed that calves in the Holstein calves exhibited higher levels of *Aspergillus* than those in the GXH group. Moreover, using LDA analysis, we obtained significantly different taxa, *Mortierella,* in Holstein cattle. Li et al. [56] found a high abundance of *Mortierella* in calves with diarrhea. Additionally, *Nigrospora*, which was significantly different in abundance in the GXH group, was found to be more abundant in healthy individuals [57]. Thus, the structure of the fungal community affects host immunity and disease resistance [58].

Fungi and bacteria coexist in the animal intestine and interact reciprocally [59]. The observations of our fungal–bacterial correlations in different breeds suggest that there are differences in kingdom interactions. Breed-specific patterns in the international network of kingdoms were noted by Spearman correlation analysis. The number and strength of fungal–bacterial correlations were much higher in GXH calves than in Holstein. On the other hand, previous studies have found weaker correlations between fungi and bacteria in the intestines of individuals more susceptible to diarrhea, and limited biodiversity in bacteria and fungi has been associated with new kingdom interactions that may be involved in inflammatory processes [60]. In contrast, the weaker correlation between gut fungi and bacteria in Holstein suggested a disconnect between the two kingdoms. Thus, the greater susceptibility of Holstein calves to diarrhea may be associated with fewer gut microbiota interactions.

## 5. Conclusions

In conclusion, this study comprehensively reported the diversity and composition of fecal microbiota of healthy calves from different breeds and showed that breeds did not alter the diversity of bacteria and fungi in calves, but there were differences in the network between bacterial–fungal kingdoms. Bacteria and fungi can communicate through signaling molecules and produce immune responses capable of modulating. This indicated that intestinal microbiota and their interactions can influence the diarrhea rate in calves. We also observed a large number of bacterial–bacterial and bacterial–fungal synergistic interactions in GXH calves. From an intestinal microbial perspective, targeting intestinal bacterial communities with interventions such as probiotics, prebiotics, antifungals, or fecal fungal transplants can improve calf intestinal health. Further, well-designed studies are needed to test this hypothesis.

## Figures and Tables

**Table 1:** The nature of the composition of concentrate (%).

Ingredient	Content
Corn	55.200
Soybean meal	18.500
Corn gluten meal	10.000
DGGS	13.000
Limestone	1.800
NaCl	0.500
Premix ^1^	1.000
Total	100.000

^1^ Premix provides the following per kg of the starter diet: VA 15,000 IU, VD 5000 IU, VE 50.000 mg, Fe 90.000 mg, Cu 12.500 mg, Mn 30.000 mg, Zn 90.000 mg, Se 0.300 mg, I 1.000 mg, Co 0.500 mg.

**Table 2:** Effect of breed on birth traits and diarrhea rate in calves.

	Body Height (cm)	Body Oblique Length (cm)	Tube Circumference (cm)	Body Weight (kg)	Diarrhea Rate (%)
GXH	78.70 ± 2.36 ^a^	77.70 ± 2.54 ^a^	9.10 ± 1.79	43.30 ± 5.48 ^a^	11.42% (4/35)
Holstein	75.70 ± 1.95 ^b^	74.00 ± 2.31 ^b^	8.00 ± 1.33	37.20 ± 5.12 ^b^	22.85% (8/35)

Note: Each value is the mean value of 10 replicates. Values are expressed as means ± SEM. ^a,b^ Means in the same column with no common superscripts differ (*p* < 0.05).

## Data Availability

The datasets generated for this study can be found in the NCBI Sequence Read Archive under BioProject PRJNA944386.

## Supplementary Materials

The following supporting information can be downloaded at: https://www.mdpi.com/article/10.3390/microorganisms12030486/s1, Table S1: Bacterial biomarkers and relative abundance identified by LEfSe in the feces of different calf breeds; Table S2: Fungal biomarkers and relative abundance identified by LEfSe in the feces of different calf breeds.
